# The role of psychological capital as a mediator in the relationship between school belonging and professional commitment among undergraduate nursing students

**DOI:** 10.3389/fpsyg.2025.1702516

**Published:** 2026-01-06

**Authors:** Yuting Lan, Xuenong Gao, Huaiyan Liu

**Affiliations:** 1 Department of Gynecology, Affiliated Renhe Hospital of China Three Gorges University, Yichang, China; 2 Teaching Management Office, Affiliated Renhe Hospital of China Three Gorges University, Yichang, China

**Keywords:** mediation, professional commitment, psychological capital, school belonging, undergraduate nursing students

## Abstract

**Objective:**

This study investigated the relationships among school belonging, psychological capital and professional commitment among nursing undergraduates, and further assessed the mediating role of psychological capital in the link between school belonging and professional commitment.

**Methods:**

A total of 301 nursing undergraduates from two universities in Hubei Province, China, participated in this cross-sectional investigation. Validated questionnaires were used to measure psychological capital, school belonging, and professional commitment. Data analysis involved Pearson correlation coefficients and mediation analyses conducted via PROCESS Model 4.

**Results:**

Findings revealed positive correlations of psychological capital and school belonging with professional commitment, and school belonging was positively associated with psychological capital. Additionally, psychological capital was found to partially mediate the relationship between school belonging and professional commitment.

**Conclusion:**

The findings suggest that improving students’ perceptions of belonging to their institution and enhancing their psychological capital could contribute to increased professional commitment among nursing undergraduates. Future longitudinal research is encouraged to verify the directionality of these relationships.

## 1 Introduction

High turnover rates and nursing workforce shortages have emerged as global challenges (Ren et al., 2024; Hu et al., 2022), with undergraduate nursing students representing the backbone of the future nursing workforce. Increased professional commitment among nurses is associated with enhanced job satisfaction (Lu et al., 2019), reduced burnout (Chang et al., 2017), and lower turnover intentions (Chang et al., 2019). Professional commitment, originating from the theories of organizational and occupational commitment, pertains to an individual’s sense of identity with their profession, their willingness to invest effort, and a favorable attitude toward continuing in that profession (Lian et al., 2005). Existing literature suggests that professional commitment typically emerges and strengthens during academic training, and the extent of such commitment among undergraduate nursing students strongly forecasts their continuation within the nursing field after graduation (Lu et al., 2000). Thus, promoting professional commitment in nursing students is crucial for ensuring workforce stability and supporting the progression of the nursing profession. However, current undergraduate nursing students face considerable academic pressure (Liu and Fu, 2024), anxiety, depression (Aloufi et al., 2021), and low professional identity (Li et al., 2023) during their studies, all of which directly undermine their professional commitment.

School belonging refers to students’ identification with their school, including their emotional experiences and attitudinal perceptions regarding interactions within the school environment (Cui et al., 2025). Baumeister proposed that belonging is a fundamental human motivation (Baumeister and Leary, 1995), critically shaping individuals’ value identification and professional goal-setting (Lee and Huang, 2021). For undergraduate nursing students, whose primary responsibility involves academic learning, the school environment significantly influences their daily experiences. A strong sense of school belonging may enhance their commitment to the nursing profession and motivate greater academic investment. Li and Shi (2021) further demonstrated a positive association between school belonging and college students’ professional commitment.

Psychological capital refers to a positive psychological state of development characterized by four core dimensions (Luthans et al., 2018): hope, self-efficacy, resilience, and optimism. It significantly influences psychological well-being (Parviniannasab et al., 2022), learning engagement (Zou et al., 2024), innovative behavior (Kotb et al., 2024), and professional identity (Feng et al., 2024). For undergraduate nursing students, psychological capital plays a crucial role in their personal and professional development, enabling them to manage academic and life challenges more effectively and enhancing their adaptability. The level of psychological capital substantially affects students’ performance in both academic learning and clinical practice. Moreover, students with higher psychological capital demonstrate stronger commitment to their professional goals.

Numerous factors impact the level of professional commitment in undergraduate nursing students. According to Bandura’s Social Cognitive Theory (Bandura, 1978), there is a reciprocal interaction among environmental contexts, personal psychological resources, and behavioral outcomes. Based on this theoretical perspective, the current study proposes a behavioral outcome framework in which school belonging represents an environmental variable, psychological capital acts as a personal psychological resource, and professional commitment is the outcome variable. School belonging describes students’ emotional bond and sense of identity with their educational institutions, serving as a significant source of environmental support. Prior studies have consistently identified school belonging as a critical environmental determinant of students’ academic behaviors and career intentions (Burger, 2023). Students who perceive stronger acceptance and recognition from their institution are generally more inclined to commit themselves to their academic pursuits and invest substantial effort. Such positive environmental influences are essential for fostering and sustaining professional commitment. Based on these considerations, this study proposes Hypothesis H1: School belonging is positively correlated with professional commitment.

Beyond this direct effect, a deeper question arises: through what internal mechanism does school belonging influence professional commitment? According to triadic reciprocal determinism, environmental influences affect behavior through individual cognition, suggesting that psychological capital may play a key mediating role. Psychological capital comprises a set of positive psychological resources developed throughout individual growth. As a measurable and cultivable internal strength, psychological capital can motivate individuals to adopt and maintain positive behaviors to achieve their goals. First, as a positive personal resource, students with higher psychological capital are better able to cope with challenges and academic pressure, sustain learning motivation (Liu et al., 2024), and consequently exhibit stronger professional commitment. Thus, Hypothesis H2 is proposed: Psychological capital is positively correlated with professional commitment.

Second, a positive school environment provides fertile ground for cultivating psychological capital. Social Cognitive Theory posits that environmental conditions shape individuals’ beliefs and self-perceptions. When students experience a strong sense of belonging, the perception of “I am accepted and valued as part of the school” can be internalized as a stable psychological strength. Therefore, this study proposes Hypothesis H3: School belonging is positively associated with psychological capital.

Moreover, school belonging can function as an environmental facilitator that promotes psychological resources such as optimism, resilience, self-efficacy, and hope. These psychological attributes subsequently enhance professional commitment by fostering students’ confidence in their potential to succeed within the nursing profession and by maintaining their motivation in the face of obstacles. This theoretical rationale underpins the mediating function of psychological capital in linking school belonging to professional commitment. Consequently, Hypothesis H4 is formulated: School belonging was positively associated with professional commitment through the mediating role of psychological capital.

## 2 Methods

### 2.1 Study design

The research utilized a descriptive cross-sectional design.

### 2.2 Participants

A convenience sampling method was employed to recruit undergraduate nursing students from two universities in Hubei Province from February to April 2025. Inclusion criteria were: (1) full-time undergraduate nursing students (freshman to senior), and (2) voluntary participation with informed consent. Exclusion criteria were: (1) students on leave during data collection. Following Kendall’s (Preacher and Kelley, 2011) sample size estimation guideline, a minimum of 5–10 observations per independent variable is recommended. Given 19 independent variables and structural equation modeling requirements (Wu, 2010) (minimum *n* = 200), along with an anticipated 20% dropout rate, the target recruitment was set at 301 participants.

### 2.3 Instruments

#### 2.3.1 General information questionnaire

Based on existing research, a self-developed questionnaire was created to collect background and demographic information. Items included were gender, academic year, student leadership experience, hometown location, whether participants were an only child, perceived employment stress, academic pressure, clinical clerkship experience, and whether nursing was initially preferred as their major.

#### 2.3.2 School belonging

The evaluation of students’ school belonging employed the School Belonging Scale developed by Chinese scholar Hao (2008). This scale contains 34 items distributed across six dimensions: recognition by the school, identification with one’s role, peer relationships, personal status, sense of responsibility, and security. Participants provided ratings on a 5-point Likert scale, where higher total scores indicated a greater sense of school belonging. The reliability coefficient (Cronbach’s α) for this scale was 0.993.

#### 2.3.3 Positive psychological capital

To evaluate the construct of psychological capital, this research adopted the Psychological Capital Scale developed by Zhang et al. (2010), a Chinese scholar. The instrument consists of four domains, hope, optimism, resilience, and self-efficacy, and includes a total of 26 items. Participants provided responses on a seven-point Likert scale, where elevated scores indicated greater psychological capital. The reliability of this instrument was demonstrated to be excellent (Cronbach’s α = 0.90).

#### 2.3.4 Nursing professional commitment

The Professional Commitment Scale, originally developed by Taiwanese scholar Lv and Qiu (1998) specifically for nursing populations, assessed students’ professional commitment. This 34-item instrument measures four dimensions: willingness to exert professional effort, career involvement, positive professional evaluation, and professional value identification. Responses used a 4-point Likert scale, with higher scores indicating stronger professional commitment. The scale showed excellent reliability (α = 0.94).

### 2.4 Data collection

Prior to data collection, approval was secured from the director of the nursing program. A web-based questionnaire was hosted on the Wenjuanxing platform.^1^ IP address restriction (one response per address) and anonymous data collection (no identifiable information) ensured data integrity and participant anonymity. All questions were mandatory to avoid missing data. After approval, grade counselors distributed the survey via QR codes to class groups. Participation was voluntary, with implied consent through survey completion. From 315 distributed surveys, 301 valid responses were collected, after excluding questionnaires with highly repetitive answer patterns and completion times under 5 min, yielding a 95.56% response rate.

### 2.5 Ethical considerations

This study received ethical authorization from the Ethics Committee of the Affiliated Renhe Hospital of China Three Gorges University (approval code: RHLL-2025-16). Written informed consent was obtained through an explicit consent selection embedded within the distributed questionnaire.

### 2.6 Data analysis

Statistical analyses involved normality assessments and chi-square tests for data screening. Frequencies and percentages were applied to describe categorical variables, while means and standard deviations summarized continuous variables. One-way ANOVA and independent samples *t*-tests were conducted to compare professional commitment levels among student groups with different characteristics. Factors influencing professional commitment were identified through multiple linear regression analyses. Pearson correlation coefficients were computed to explore associations among school belonging, psychological capital, and professional commitment. A mediation model was constructed using Model 4 of the PROCESS component. A 95% confidence interval was established through the Bootstrap method with 5,000 repeated samples, and the significance level was set at α = 0.05.

## 3 Results

### 3.1 Comparison of professional commitment scores across demographic characteristics

The 301 undergraduate nursing students ranged in age from 17 to 27 years (20.26 ± 1.27). Among them, 64 (21.26%) were male and 237 (78.74%) female. By academic year, there were 71 (23.59%) first-year, 74 (24.58%) second-year, 84 (27.91%) third-year, and 72 (23.92%) fourth-year students. Further demographic details are presented in Table 1. Univariate analysis revealed significant variations in professional commitment scores associated with factors such as academic year, preference for nursing as the initial major, employment-related anxiety, perceived academic pressure, and clinical internship experience.

**Table 1:** Professional commitment by demographic characteristics (*n* = 301).

Variable	*N* (%)	M ± SD	*t/F*	*P*
Gender			−0.900	0.369
Male	64 (21.26)	95.89 ± 14.72		
Female	237 (78.74)	97.56 ± 12.73		
Academic year			3.073	0.028
First-year students	71 (23.59)	93.86 ± 14.16		
Second-year students	74 (24.58)	100.42 ± 10.98		
Third-year students	84 (27.91)	97.00 ± 13.11		
Fourth-year students	72 (23.92)	97.44 ± 13.72		
Hometown location			−1.187	0.236
Rural	207 (68.77)	96.6 ± 13.01		
Urban	94 (31.23)	98.54 ± 13.50		
Only-child status			−0.087	0.931
Yes	104 (34.55)	97.12 ± 14.96		
No	197 (65.45)	97.25 ± 12.16		
Student leadership experience			−0.069	0.945
Yes	121 (40.20)	97.14 ± 14.31		
No	180 (59.80)	97.25 ± 12.39		
First-choice nursing program			2.466	0.014
Yes	163 (54.15)	98.91 ± 13.31		
No	138 (45.85)	95.19 ± 12.76		
Concern about future employment			−2.043	0.048
Yes	266 (88.37)	96.52 ± 12.53		
No	35 (11.63)	102.46 ± 16.59		
Academic pressure			7.055	0.001
Low	29 (9.63)	89.66 ± 12.97		
Moderate	117 (38.87)	99.61 ± 14.25		
High	155 (51.50)	96.81 ± 11.80		
Clinical clerkship experience			4.003	<0.001
Yes	186 (61.79)	99.54 ± 13.18		
No	115 (38.21)	93.43 ± 12.31		

### 3.2 Current status of school belonging, psychological capital, and professional commitment scores

Professional commitment scores ranged from 34 to 136, with an overall moderate level (97.21 ± 13.17). Dimension scores ranked from highest to lowest were: professional effort willingness (47.59 ± 9.85), career involvement (21.04 ± 5.26), value identification (15.69 ± 2.83), and positive evaluation (12.88 ± 2.57). School belonging and psychological capital scores were 130.44 ± 21.67 and 122.57 ± 22.48, respectively (Table 2).

**Table 2:** Correlation analysis of three scales (*n* = 301).

Scale	Score range	*M* ± SD	1	2	3
1. School belonging	34–170	130.44 ± 21.67			
School recognition	12–60	46.30 ± 8.33			
Self-role recognition	5–25	18.48 ± 6.13			
Peer relationship recognition	5–25	19.67 ± 3.31			
Personal status recognition	4–20	15.62 ± 2.83			
Sense of security	4–20	15.92 ± 2.81			
Sense of responsibility	4–20	14.45 ± 3.14			
2. Psychological capital	26–182	122.57 ± 22.48	0.708^***^		
Resilience	7–49	29.44 ± 6.51			
Self-efficacy	7–49	33.50 ± 8.23			
Hope	6–42	29.75 ± 5.82			
Optimism	6–42	29.88 ± 6.66			
3. Professional commitment	34–136	97.21 ± 13.17	0.555^***^	0.497^***^	
Professional effort willingness	16–64	47.59 ± 9.85			
Professional career involvement	8–32	21.04 ± 5.26			
Professional positive evaluation	5–20	12.88 ± 2.57			
Professional value identification	5–20	15.69 ± 2.83			

*** indicates statistical significance at the *p* <0.001 level.

### 3.3 Regression analysis of factors influencing professional commitment

Multiple regression analysis was conducted, treating professional commitment total scores as the dependent variable, to identify relevant influencing factors. Independent variables that demonstrated significance in both Pearson’s correlations and univariate analyses were incorporated. According to Table 3, professional commitment scores were notably influenced by students’ year of study (β = −0.173, *p* = 0.002), nursing as the primary choice of major (β = −0.138, *p* = 0.003), concern about employment prospects (β = 0.100, *p* = 0.036), and experience with clinical internships (β = −0.203, *p* < 0.001).

**Table 3:** Predictors of professional commitment (*n* = 301).

Independent variables	*B*	β	*SE*	95% CI	*P*	VIF
Constant	60.425	–	6.583	[47.469, 73.382]	<0.001	–
Academic year	−2.072	−0.173	0.675	[−3.401, −0.744]	0.002	1.533
First-choice nursing program	−3.647	−0.138	1.209	[−6.026, −1.268]	0.003	1.018
Concern about future employment	4.120	0.100	1.952	[0.278, 7.961]	0.036	1.099
Academic pressure	1.788	0.09	0.992	[−0.165, 3.740]	0.073	1.205
Clinical clerkship experience	−5.499	−0.203	1.476	[−8.405, −2.594]	<0.001	1.444
Psychological capital	0.127	0.217	0.040	[0.049, 0.205]	0.001	2.227
School belonging	0.233	0.384	0.041	[0.153, 0.313]	<0.001	2.161

*R*^²^ = 0.396, Adjusted *R*^²^ = 0.377, *F* = 27.46, *p* <0.001 level.

### 3.4 Correlations among school belonging, professional commitment, and psychological capital

Correlation analysis (Table 2) revealed significant positive relationships among school belonging, psychological capital, and professional commitment: school belonging and professional commitment (*r* = 0.555, *p* < 0.001), school belonging and psychological capital (*r* = 0.708, *p* < 0.001), and psychological capital and professional commitment (*r* = 0.497, *p* < 0.001).

### 3.5 Mediating role of psychological capital between school belonging and professional commitment

**Figure 1:**
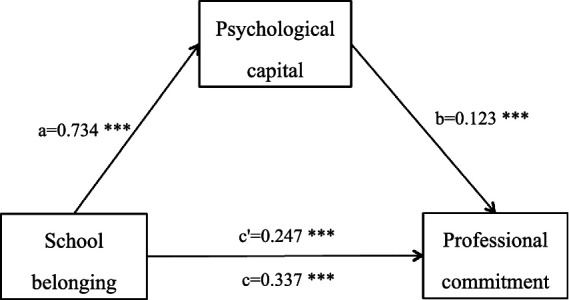
Psychological capital’s mediating role between school belonging and professional commitment. ***p < 0.001.

Mediation testing was carried out using Model 4 of PROCESS, assigning psychological capital as the mediator, school belonging as the independent variable, and professional commitment as the dependent variable. Bootstrap resampling with 5,000 iterations and 95% confidence intervals (CIs) was applied; intervals not containing zero indicated statistical significance (Preacher and Hayes, 2004). Results revealed significant positive effects of school belonging on psychological capital (β = 0.734, *p* < 0.001) and on professional commitment (β = 0.337, *p* < 0.001). Additionally, psychological capital, as the mediator, significantly predicted professional commitment (β = 0.123, *p* < 0.001), and school belonging continued to have a notable direct impact (β = 0.247, *p* < 0.001). The 95% CIs for the direct effect (β = 0.247, 95% CI: 0.167–0.328) and indirect effect (β = 0.090, 95% CI: 0.027–0.161) both excluded zero, thereby confirming psychological capital’s role as a partial mediator. Specifically, the indirect effect accounted for 26.70% (0.090) of the total observed relationship (0.337), while the direct contribution represented 73.30% (0.247) (Table 4; Figure 1).

**Table 4:** Mediating role of psychological capital in school belonging and professional commitment (*n* = 301).

Effects	Path	β	SE	95%CI	Effect percentage
Direct effect	School belonging → professional commitment (c′)	0.247	0.041	[0.167, 0.328]	73.30%
Indirect effect	School belonging → psychological capital → professional commitment (a×b)	0.090	0.084	[0.027, 0.161]	26.70%
Total effect	School belonging → professional commitment (c)	0.337	0.029	[0.280, 0.395]	100%

## 4 Discussion

### 4.1 Professional commitment among undergraduate nursing students and its influencing factors

The professional commitment of undergraduate nursing students was at a moderate level, consistent with findings by Ahmadzadeh-Zeidi et al. (2024). Among the dimensions, students scored highest in “professional effort willingness” and lowest in “professional positive evaluation.” This indicates that students are generally willing to invest effort into their professional growth but need stronger identification and positive views about nursing. Nursing educators and administrators should recognize this issue and implement targeted interventions to enhance students’ professional commitment and career willingness. The study identified several factors associated with professional commitment among undergraduate nursing students. Second-year students showed the highest professional commitment, consistent with research by Zhang et al. (2024). This may be because second-year students are at a critical stage of developing professional identity. They have a stable initial understanding of the profession but have not yet faced significant academic pressures. Moreover, they are starting core courses and clinical practice, improving both theoretical knowledge and practical skills. Students who selected nursing as their first-choice major had higher professional commitment. According to Vroom’s Career Motivation Theory (Vroom, 1964), initial career choice significantly influences students’ professional identity and development. Students with nursing as their first choice usually have a clearer understanding and stronger interest in the profession. Thus, they tend to be more resilient when facing academic and career challenges. Concerns about future employment correlated with lower professional commitment. This may relate to nursing’s demanding work intensity (Ren et al., 2022), limited career development opportunities (Liu et al., 2021), and low public recognition of the profession (Chen et al., 2025). Such factors can lead students to worry about their career prospects, reducing their professional commitment (Ayaz-Alkaya et al., 2018). Clinical clerkship experience was associated with higher professional commitment scores, supporting findings from a study among Turkish undergraduate nursing students. Clinical internships enhance students’ professional identity and competencies by immersing them in real clinical environments and engaging them in patient care early. This experiential learning strengthens their understanding of their future roles, deepening professional commitment. Nursing educators should emphasize career development education, understand students’ professional attitudes, increase early practice opportunities, and develop targeted training programs. Additionally, enhancing mental health education to alleviate employment anxiety and improving public perceptions through promotional activities can help students better appreciate the nursing profession and build a stronger professional identity.

### 4.2 Relationships among school belonging, psychological capital, and professional commitment

Students’ school belonging was at a moderate to high level, consistent with previous studies (McKenna et al., 2013). The “school recognition” dimension had the highest score, indicating that students generally feel their academic efforts and personal values are acknowledged, strengthening their belonging. However, the “sense of responsibility” dimension had the lowest scores, which warrants attention. The sense of responsibility reflects students’ identification with and willingness to contribute to their school. Lower scores in this dimension may result from issues related to curriculum design, campus culture, or student-faculty interactions. Correlation analyses demonstrated a positive association between school belonging and professional commitment, consistent with prior studies such as those conducted by Li and Shi (2021). Students with a heightened sense of school belonging exhibited stronger professional identification, enhanced academic motivation, and clearer vocational objectives. Such positive psychological states help students maintain commitment and consistency in professional learning, crucial for improving nursing education quality. Therefore, nursing educators should promote collective activities, enhance campus culture, and encourage volunteer service to increase students’ sense of responsibility.

Levels of psychological capital among nursing students were observed to be moderate to high, aligning with previous research outcomes (Terry et al., 2019). The dimension of “self-efficacy” received the highest score, indicating that nursing students possess considerable confidence in managing academic responsibilities. Conversely, the lowest scores were observed in “resilience,” suggesting that certain students may need additional support to strengthen their stress-coping abilities, particularly in response to academic and professional challenges. Additionally, psychological capital exhibited a significantly positive correlation with professional commitment, in alignment with findings from Yu et al. (2023). Higher psychological capital correlated with more favorable professional attitudes, including stronger commitment to learning, clearer professional identification, and well-defined career ambitions. Self-efficacy, a fundamental component of psychological capital, is influential in shaping students’ decisions and effort exerted toward academic and professional tasks. Students with strong self-efficacy adopt more active learning strategies and show greater resilience and problem-solving ability when facing difficulties (Altıntaş et al., 2024). It is suggested that nursing educators focus on cultivating students’ self-efficacy, practical skills, and career planning awareness to enhance their psychological capital.

### 4.3 The partial mediating role of psychological capital between school belonging and professional commitment

The findings show that school belonging was positively associated with professional commitment, with psychological capital partially mediating this relationship. A strong sense of school belonging was linked to higher psychological capital, and higher psychological capital was linked to greater professional commitment. Bandura’s Social Cognitive Theory states that the environment, the individual, and behavior interact dynamically. School belonging, as a key environmental factor, reflects how well students identify with and integrate into the school. It directly influences their learning attitudes and behaviors. Research in Australia has shown that school belonging is an important indicator of whether nursing students can successfully complete their studies (Middleton et al., 2021). Albloushi et al. (2019) also reported that students with a stronger sense of belonging adapt better to the clinical and campus environment, which strengthens motivation and professional identity. Beyond the direct effect, school belonging can indirectly influence professional commitment through psychological capital. Woo and Park’s (2017) study found that higher psychological capital improves professional satisfaction. Students who feel supported and recognized by their institution tend to develop greater confidence, maintain optimism, and persist more effectively through challenges. These psychological strengths contribute to higher psychological capital, which further reinforces their belief in building meaningful nursing careers. Therefore, it is recommended that nursing educators help students enhance psychological capital through mental health education, career planning guidance, team-building activities, and alumni engagement. At the same time, when teachers provide academic and emotional support, when parents participate actively, and when counselors and administrators strengthen school relationships and leadership, students’ sense of belonging increases (Turan Bora and Akbaba Altun, 2025), thereby further improving their paths toward professional commitment.

## 5 Limitations and future research

There are several limitations of this investigation that should be recognized. Firstly, due to the cross-sectional methodology employed, causal interpretations regarding the studied variables cannot be conclusively established. Secondly, the reliance on convenience sampling involving participants from merely two universities within a single geographic region restricts the external validity and applicability of the results. Therefore, subsequent studies should employ longitudinal or experimental methodologies and involve broader and more representative samples to verify the causal directions and enhance the generalizability of the observed associations.

Despite these limitations, the findings offer a compelling impetus for further research. Given the established roles of psychological capital and school belonging, researchers should develop targeted interventions. For example, programs that enhance psychological capital, such as resilience training, mentorship initiatives, and simulation-based exercises promoting self-efficacy, could be systematically implemented and evaluated. Similarly, interventions aimed at increasing school belonging, including team-building activities, volunteer programs, and structured alumni engagement, need rigorous testing. Future studies should evaluate the feasibility and effectiveness of such interventions, which would help determine whether they can meaningfully improve professional commitment, academic performance, and career intentions.

## 6 Conclusions and implications

This study revealed a positive association between school belonging and professional commitment among undergraduate nursing students, with psychological capital playing a partial mediating role in this relationship. These findings hold significant theoretical and practical implications. Theoretically, this study reveals the relationships among school belonging, psychological capital, and professional commitment, providing a theoretical basis for subsequent research. In addition, by exploring the mediating effect, it deepens the understanding of the formation mechanism of professional commitment. Meanwhile, the method adopted in this study offers a reference for similar research and contributes to promoting the in-depth development of quantitative research in the field of nursing education. Practically, this study provides new insights into enhancing the professional commitment of nursing undergraduates. Firstly, given the positive impact of school belonging on psychological capital and professional commitment, universities can strengthen students’ sense of school identification and emotional bonds by optimizing campus culture, expanding student club activities, and enhancing teacher-student interaction. Secondly, nursing educators should emphasize cultivating students’ psychological capital. For instance, they can offer mental health courses, organize group psychological counseling, conduct positive psychological training, and provide personalized development guidance to help students establish a positive self-perception, improve psychological resilience, and cultivate an optimistic attitude and a goal-oriented sense of hope. In conclusion, nursing managers and educators can take two approaches—strengthening school belonging and developing psychological capital—to improve the professional commitment of undergraduate nursing students, thereby promoting the stability and sustainable development of the nursing workforce.

## Data Availability

The raw data supporting the conclusions of this article will be made available by the authors, without undue reservation.

## Footnotes

1
https://www.wjx.cn
