# Correction: The role of psychological capital as a mediator in the relationship between school belonging and professional commitment among undergraduate nursing students

**DOI:** 10.3389/fpsyg.2026.1785610

**Published:** 2026-02-02

**Authors:** Yuting Lan, Xuenong Gao, Huaiyan Liu

**Affiliations:** 1 Department of Gynecology, Affiliated Renhe Hospital of China Three Gorges University, Yichang, China; 2 Teaching Management Office, Affiliated Renhe Hospital of China Three Gorges University, Yichang, China

**Keywords:** mediation, professional commitment, psychological capital, school belonging, undergraduate nursing students

There was a mistake in Figure 1 as published. The text labels were presented in red, which may cause visual accessibility issues when printed in black and white. The corrected Figure 1 appears below.

**Figure 1:**
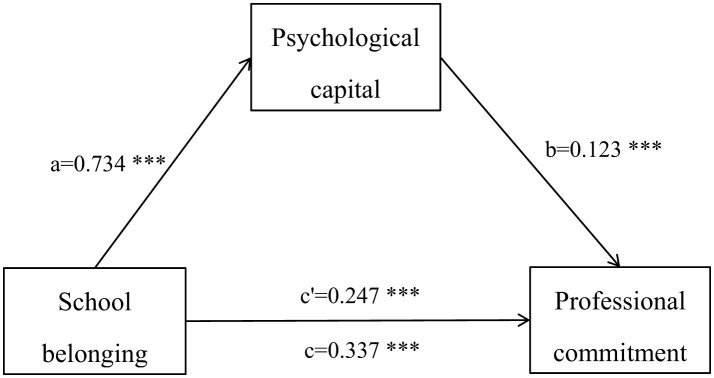
Psychological capital's mediating role between school belonging and professional commitment. ^***^p < 0.001.

There was a mistake in Table 4 as published. The “Total effect” row incorrectly labeled the path as (c′) instead of the standard notation (c). The corrected Table 4 appears below.

**Table 4:** Mediating role of psychological capital in school belonging and professional commitment (*n* = 301).

**Effects**	**Path**	**β**	**SE**	**95% CI**	**Effect percentage**
Direct effect	School belonging → professional commitment (c′)	0.247	0.041	[0.167, 0.328]	73.30%
Indirect effect	School belonging → psychological capital → professional commitment (a × b)	0.090	0.084	[0.027, 0.161]	26.70%
Total effect	School belonging → professional commitment (c)	0.337	0.029	[0.280, 0.395]	100%

The original version of this article has been updated.

